# A Phase Field Approach to Two-Dimensional Quasicrystals with Mixed Mode Cracks

**DOI:** 10.3390/ma16103628

**Published:** 2023-05-09

**Authors:** Tong Li, Zhenting Yang, Chenghui Xu, Xinsheng Xu, Zhenhuan Zhou

**Affiliations:** 1 State Key Laboratory of Structural Analysis, Optimization and CAE Software for Industrial Equipment, Department of Engineering Mechanics, International Center for Computational Mechanics, Dalian University of Technology, Dalian 116024, China; 2 School of Mechanics, Civil Engineering and Architecture, Northwestern Polytechnical University, Xi’an 710072, China

**Keywords:** phase field model, decagonal quasicrystal, crack propagation, brittle fracture, mixed mode crack, finite element method

## Abstract

Quasicrystals (QCs) are representatives of a novel kind of material exhibiting a large number of remarkable specific properties. However, QCs are usually brittle, and crack propagation inevitably occurs in such materials. Therefore, it is of great significance to study the crack growth behaviors in QCs. In this work, the crack propagation of two-dimensional (2D) decagonal QCs is investigated by a fracture phase field method. In this method, a phase field variable is introduced to evaluate the damage of QCs near the crack. Thus, the crack topology is described by the phase field variable and its gradient. In this manner, it is unnecessary to track the crack tip, and therefore remeshing is avoided during the crack propagation. In the numerical examples, the crack propagation paths of 2D QCs are simulated by the proposed method, and the effects of the phason field on the crack growth behaviors of QCs are studied in detail. Furthermore, the interaction of the double cracks in QCs is also discussed.

## 1. Introduction

Quasicrystals (QCs) are a new kind of material with perfect long-range order but no periodicity. Due to their unique atomic structures, QCs exhibit many excellent physical properties, such as high hardness, a low friction coefficient, and high resistance. Therefore, QCs are very promising materials for potential applications in corrosion-resistant coatings, hydrogen storage, photovoltaic solar cells, etc. [1]. However, QCs are usually brittle, and consequently, cracks, holes, and other defects will inevitably occur during daily use. If the crack propagates, the QC will fail, which may lead to a catastrophic accident. Therefore, the fracture analysis of QCs is of great importance, and it is very necessary to investigate crack propagation behaviors in cracked QCs.

Plenty of research work has been carried out on the fracture problems of QCs. Li et al. [2] extended the classical linear elasticity fracture mechanics to investigate a decagonal QC with a Griffith crack. The results indicated the phonon and phason stresses at the crack tip exhibit the well-known square root singularity, and the strain energy release rate was obtained. Zhou and Fan [3] developed the plane elasticity theory of two-dimensional (2D) octagonal QCs and obtained the exact analytic solution of a Mode I Griffith crack. Guo and Fan [4] studied the fracture problem of a Mode II Griffith crack in decagonal quasicrystals and obtained the corresponding stress intensity factors and strain energy release rate. Shen and Fan [5] calculated the stress intensity factors for an infinitely long strip of finite height containing two straight, semi-infinite collinear cracks. Li and Fan [6] obtained exact solutions for two semi-infinite collinear cracks in a strip of 1D hexagonal QCs. After that, they [7] further obtained an analytic solution for the elliptic notch problem of the material in icosahedral QCs by using the complex variable method. The solution can be reduced to that of a Griffith crack problem. Fan et al. [8] presented linear, nonlinear, and dynamic fracture problems for different QCs. Li and Liu [9] obtained closed-form expressions for the elastic displacement and stress fields induced by a dislocation in icosahedral QCs. Sladek et al. [10] developed a meshless method based on the local Petrov-Galerkin approach for fracture analysis of decagonal QCs; both static and transient dynamic boundary value problems were considered. After that, they [11] present path-independent integrals for accurate evaluation of energy release and stress intensity factors in decagonal QCs. Wang et al. [12] obtained the phonon-phason coupling field in the half-space, which can be expressed in terms of elementary functions. These solutions could have applications in 3D contact and crack problems in QCs. Li et al. [13,14] derived solutions for elliptical crack and planar crack problems in 2D hexagonal QCs. Li and Shi [15] employed the method of potential function theory to solve plane defect problems originating from two-dimensional decagonal QCs. Zhao et al. [16] derived the fundamental solutions for interface cracks in 1D hexagonal QC coatings under in-plane loads.

For crack propagation in QCs, some investigations have been conducted based on the atomistic model [17,18,19,20,21,22,23,24,25,26,27,28,29]. However, it is inconvenient to apply atomistic simulation to engineering. Therefore, Wang and Ricoeur [30] adopted the finite element method (FEM) to simulate the crack growth in 1D QCs and predicted the crack pattern for different boundary conditions. Fan et al. [31,32,33,34,35,36,37,38,39] investigated crack propagation based on the elastodynamic/hydrodynamic model.

From the above literature review, it is found that increasing attention has been paid to the fracture of QCs. However, most of them were concentrated on the derivation of exact solutions and determination of fracture parameters of cracked QCs, and the investigations on crack propagation were mainly based on the atomistic model [17,18,19,20,21,22,23,24,25,26,27,28,29]. The work on crack growth in QCs based on continuum mechanics is still insufficient. Additionally, in the traditional FEM, the crack topology is modeled by geometry. The remesh process is necessary when simulating crack propagation, which has a huge computational cost. In this paper, a phase field method is introduced to predict the crack propagation path in QCs. Unlike conventional discrete crack models (such as the FEM), the fracture phased field method employs diffusive cracks to avoid an explicit representation of kinematic discontinuities, and therefore the propagating cracks are tracked automatically without additional ad-hoc criteria in the classical Griffith fracture theory [40,41]. In this method, the fracture energy and degraded strain energy of QCs are formulated using the phase field variable. Subsequently, the total potential energy of QCs under the phase field framework is obtained, and the governing equations for the phase field model are derived by means of the Francfort-Marigo variational principle. The phase field evolution equation for QCs is constructed. Finally, the FEM is adopted to solve the phase field governing equations. The phase field variable and phonon/phason displacement of the entire model can be obtained, as well as the reaction force and the crack pattern.

This paper is organized as follows: The basic equations of 2D decagonal QCs are presented in Section 2.1. The phase field model for 2D QCs is formulated in Section 2.2. The finite element implementation for the phase field model is presented in Section 2.3. Several numerical examples are presented in Section 3. Conclusions are summarized in Section 4.

## 2. Phase Field Method for 2D Decagonal QCs

### 2.1. The Basic Equations

According to the elasticity of 2D decagonal QCs, the basic equations for the plane problem of QCs in the absence of body force and phason self-action are [42]:

(1) $$\left\{\begin{array}{c} \frac{\partial \sigma_{x}}{\partial x}+\frac{\partial \tau_{xy}}{\partial y}=0\\ \frac{\partial \tau_{yx}}{\partial x}+\frac{\partial \sigma_{y}}{\partial y}=0 \end{array}\right.,\;\left\{\begin{array}{c} \frac{\partial H_{x}}{\partial x}+\frac{\partial H_{xy}}{\partial y}=0\\ \frac{\partial H_{yx}}{\partial x}+\frac{\partial H_{y}}{\partial y}=0 \end{array}\right.$$

(2) $$\left\{\begin{array}{c} \varepsilon_{x}\\ \varepsilon_{y}\\ \gamma_{xy} \end{array}\right\}=\left[\begin{array}{cc} \frac{\partial }{\partial x} & \\ & \frac{\partial }{\partial y}\\ \frac{\partial }{\partial y} & \frac{\partial }{\partial x} \end{array}\right]\left\{\begin{array}{c} u_{x}\\ u_{y} \end{array}\right\},\;\left\{\begin{array}{c} \omega_{x}\\ \omega_{y}\\ \omega_{xy}\\ \omega_{yx} \end{array}\right\}=\left[\begin{array}{cc} \frac{\partial }{\partial x} & \\ & \frac{\partial }{\partial y}\\ \frac{\partial }{\partial y} & \\ & \frac{\partial }{\partial x} \end{array}\right]\left\{\begin{array}{c} w_{x}\\ w_{y} \end{array}\right\}$$

(3) $$\left\{\begin{array}{c} \sigma_{x}\\ \sigma_{y}\\ \tau_{xy} \end{array}\right\}=\left[\begin{array}{ccc} C_{11} & C_{12} & \\ C_{12} & C_{22} & \\ & & C_{66} \end{array}\right]\left\{\begin{array}{c} \varepsilon_{x}\\ \varepsilon_{y}\\ \gamma_{xy} \end{array}\right\}+\left[\begin{array}{cccc} R_{1} & R_{1} & R_{2} & -R_{2}\\ -R_{1} & -R_{1} & -R_{2} & R_{2}\\ R_{2} & R_{2} & -R_{1} & R_{1} \end{array}\right]\left\{\begin{array}{c} \omega_{x}\\ \omega_{y}\\ \omega_{xy}\\ \omega_{yx} \end{array}\right\}$$

(4) $$\left\{\begin{array}{c} H_{x}\\ H_{y}\\ H_{xy}\\ H_{yx} \end{array}\right\}=\left[\begin{array}{ccc} R_{1} & -R_{1} & R_{2}\\ R_{1} & -R_{1} & R_{2}\\ R_{2} & -R_{2} & -R_{1}\\ -R_{2} & R_{2} & R_{1} \end{array}\right]\left\{\begin{array}{c} \varepsilon_{x}\\ \varepsilon_{y}\\ \gamma_{xy} \end{array}\right\}+\left[\begin{array}{cccc} K_{1} & K_{2} & & \\ K_{2} & K_{1} & & \\ & & K_{1} & -K_{2}\\ & & -K_{2} & K_{1} \end{array}\right]\left\{\begin{array}{c} \omega_{x}\\ \omega_{y}\\ \omega_{xy}\\ \omega_{yx} \end{array}\right\}$$

where $u_{x}$ and $u_{y}$ are the phonon displacements; $w_{x}$ and $w_{y}$ are the phason displacements; $\varepsilon_{x}$, $\varepsilon_{y}$, and $\gamma_{xy}$ are the phonon strains; $\omega_{x}$, $\omega_{y}$, $\omega_{xy}$, and $\omega_{yx}$ are the phason strains; $\sigma_{x}$, $\sigma_{y}$, and $\sigma_{xy}$ are the phonon stresses; $H_{x}$, $H_{y}$, $H_{xy}$, and $H_{yx}$ are the phason stresses; $C_{11}$, $C_{12}$, $C_{22}$, and $C_{66}$ are the phonon moduli; $K_{1}$ and $K_{2}$ are the phason moduli; and $R_{1}$ and $R_{2}$ are the phonon-phason coupling coefficients.

The strain energy of QCs is:

(5) $${\mathrm{II}}_{s}=\int_{\Omega }^{}\psi \left(\varepsilon ,\;\omega \right)\;dV=\int_{\Omega }^{}\frac{1}{2}\varepsilon^{T}\sigma +\frac{1}{2}\omega^{T}H\;dV$$

where $\sigma ={\left\{\sigma_{x},\;\sigma_{y},\;\sigma_{xy}\right\}}^{T}$ and $H={\left\{H_{x},\;H_{y},\;H_{xy},\;H_{yx}\right\}}^{T}$ are the stress vectors; $\varepsilon ={\left\{\varepsilon_{x},\;\varepsilon_{y},\;\gamma_{xy}\right\}}^{T}$ and $\varepsilon ={\left\{\omega_{x},\;\omega_{y},\;\omega_{xy},\;\omega_{yx}\right\}}^{T}$ are the strain vectors; and $\psi \left(\varepsilon ,\;\omega \right)$ is the strain energy density.

### 2.2. Phase Field Method

**Figure 1:**
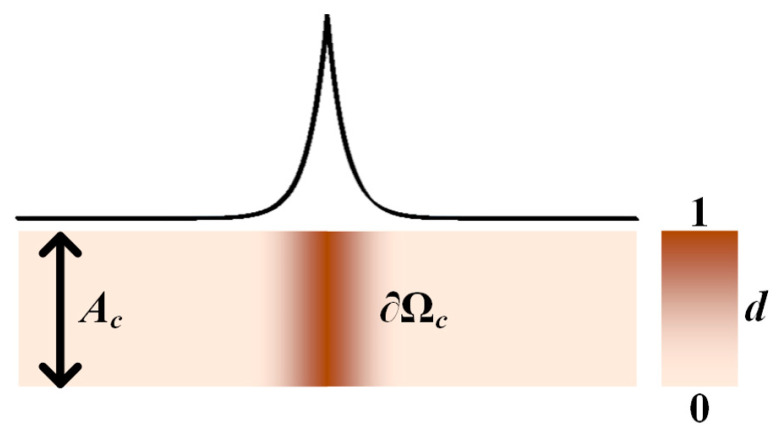
A QC strip with a centered crack.

Consider a 1D QC strip with a centered crack, as shown in Figure 1. The fracture energy is:

(6) $${\mathrm{II}}_{c}=\int_{\partial \Omega_{c}}G_{c}\;dS=G_{c}A_{c}$$

where $G_{c}$ is the critical energy release rate (CERR); $\partial \Omega_{c}$ is the crack surface; and $A_{c}$ is the cross-sectional area of the strip.

In a phase field model, the crack is supposed to be surrounded by a diffusive degraded zone, and a phase field variable *d* is introduced to describe the damage in the diffusive degraded zone:

(7) $$d=e^{-\frac{\left|x\right|}{l_{c}}}$$

where *l_c_* is an internal length scale that controls the width of the diffusive zone. Here, it is noted that, when *x* approaches infinity (±∞), *d* converges to zero, which indicates that the material is intact; when *x* is zero, *d* equals one, which indicates that the material is totally broken and the crack surface is produced. Define a crack’s surface density as functional [43]:

(8) $$\gamma \left(d,\;d^{\prime }\right)=\frac{1}{2l_{c}}\left(d^{2}+l_{c}^{2}{d^{\prime }}^{2}\right)$$

The crack topology can then be described by the phase field variable.

Substituting Equation (8) into Equation (6) yields the fracture energy in the phase field model:

(9) $${\mathrm{II}}_{c}=\int_{\Omega }G_{c}\gamma \left(d,\;d^{\prime }\right)\;dV$$

where Ω is the domain of the overall model. As observed in Equation (9), the integral over the crack path is transformed into the integral over the model. The crack topology is implicitly expressed in the framework of the phase field method. Therefore, it is unnecessary to track the crack’s path while it is propagating.

Similarly, the 2D crack surface density functional can be defined as:

(10) $$\gamma \left(d,\;\nabla d\right)=\frac{1}{2l_{c}}\left(d^{2}+l_{c}^{2}{\left|\nabla d\right|}^{2}\right)$$

Therefore, the fracture energy of QCs in a plane problem is:

(11) $${\mathrm{II}}_{c}=\int_{\Omega }G_{c}\gamma \left(d,\;\nabla d\right)\;dV$$

In the phase field model, the material at the crack is assumed to be softened. Therefore, a degradation function is introduced to evaluate the damage to the material in the diffusive degraded zone [43]:

(12) $$\omega \left(d\right)={\left(1-d\right)}^{2}+k$$

where *k* is a small positive number to ensure the non-singularity of the matrix. It can be observed that $\omega \left(d\right)$ satisfies $\omega \left(0\right)=1$ and $\omega \left(1\right)=0$.

The strain energy for QCs is modified as follows:

(13) $${\mathrm{II}}_{s}=\int_{\Omega }^{}\omega \left(d\right)\psi \left(\varepsilon ,\;\omega \right)\;dV$$

It should be pointed out that the crack will not propagate if the crack face is under compression. Therefore, the strain energy density ψ should be decomposed into a tensile part and a compressed part, and the energy degradation only occurs on the tensile part [43]:

(14) $${\mathrm{II}}_{s}=\int_{\Omega }^{}\omega \left(d\right)\psi_{+}+\psi_{-}\;dV$$

where $\psi_{+}=\lambda {\langle \varepsilon_{1}+\varepsilon_{2}+\varepsilon_{3}\rangle }_{+}^{2}/2+\mu \left[{\langle \varepsilon_{1}\rangle }_{+}^{2}+{\langle \varepsilon_{2}\rangle }_{+}^{2}+{\langle \varepsilon_{3}\rangle }_{+}^{2}\right]$ and $\psi_{-}=\psi -\psi_{+}$ are the tensile and compressed strain energy densities, respectively; ${\langle \rangle }_{+}$ is defined as ${\langle x\rangle }_{+}=\left(\left|x\right|+x\right)/2$; and λ and μ are the Lamé constants.

The potential energy of the overall model contains the strain energy, fracture energy, and potential energy of the external force:

(15) $$\begin{array}{ll} {\mathrm{II}}_{p} & ={\mathrm{II}}_{s}+{\mathrm{II}}_{c}-{\mathrm{II}}_{e}\\ & =\int_{\Omega }^{}\omega \left(d\right)\psi_{+}+\psi_{-}\;dV+\int_{\Omega }G_{c}\gamma \left(d,\;\nabla d\right)\;dV\\ & -\int_{\partial \Omega }u^{T}t^{u}\;dS-\int_{\partial \Omega }w^{T}t^{w}\;dS \end{array}$$

where $u={\left\{u_{x},\;u_{y}\right\}}^{T}$; $w={\left\{w_{x},\;w_{y}\right\}}^{T}$; and $t^{u}$ and $t^{w}$ are the external forces in the phonon and phason fields, respectively.

The Francfort-Marigo variational principle states that the real displacement **u** and the phase field variable *d* will minimize the potential energy.

(16) $$\begin{array}{ll} \delta {\mathrm{II}}_{p} & =\int_{\partial \Omega_{u}}^{}\omega \left(d\right)\sigma_{ij}^{+}n_{i}\delta u_{j}+\sigma_{ij}^{-}n_{i}\delta u_{j}\;dS+\int_{\partial \Omega_{w}}^{}\omega \left(d\right)H_{ij}^{+}n_{i}\delta w_{j}+H_{ij}^{-}n_{i}\delta w_{j}\;dS\\ & -\int_{\Omega }^{}\frac{\partial \left[\omega \left(d\right)\sigma_{ij}^{+}\right]}{\partial x_{i}}\delta u_{j}+\frac{\partial \sigma_{ij}^{-}}{\partial x_{i}}\delta u_{j}\;dV-\int_{\Omega }^{}\frac{\partial \left[\omega \left(d\right)H_{ij}^{+}\right]}{\partial x_{i}}\delta w_{j}+\frac{\partial H_{ij}^{-}}{\partial x_{i}}\delta w_{j}\;dV\\ & \int_{\Omega }^{}\omega^{\prime }\left(d\right)\psi_{+}\delta d\;dV+\int_{\Omega }\frac{G_{c}}{l_{c}}d\delta d\;dV+\int_{\partial \Omega }G_{c}l_{c}\frac{\partial d}{\partial x_{i}^{}}n_{i}\delta d\;dS-\int_{\Omega }G_{c}l_{c}\frac{\partial^{2}d}{\partial x_{i}^{2}}\delta d\;dV\\ & -\int_{\partial \Omega }\delta u_{j}t_{j}^{u}\;dS-\int_{\partial \Omega }\delta w_{j}t_{j}^{w}\;dS\\ & =0 \end{array}$$

where $\sigma_{ij}^{+}$ and $H_{ij}^{+}$ are the stresses induced by stretch, while $\sigma_{ij}^{-}$ and $H_{ij}^{-}$ are induced by compression. Equation (16) is valid for arbitrary $\delta u_{i}$, $\delta w_{i}$, and δd. Hence, the governing equations for the phase field model of QCs are:

(17) $$\nabla \left[\omega \left(d\right)\sigma_{ij}^{+}+\sigma_{ij}^{-}\right]=0,\;\Omega$$

(18) $$\nabla \left[\omega \left(d\right)H_{ij}^{+}+\partial H_{ij}^{-}\right]=0,\;\Omega$$

(19) $$\left(2d-2\right)\psi_{+}+\frac{G_{c}}{l_{c}}d-G_{c}l_{c}\Delta d=0,\;\Omega$$

(20) $$\left[\omega \left(d\right)\sigma_{ij}^{+}+\sigma_{ij}^{-}\right]n_{i}=t_{j}^{u},\;\partial \Omega$$

(21) $$\left[\omega \left(d\right)H_{ij}^{+}+H_{ij}^{-}\right]n_{i}=t_{j}^{w},\;\partial \Omega$$

(22) $$d_{,i}n_{i}=0,\;\partial \Omega$$

It should be mentioned that crack growth is an irreversible process. Therefore, Equation (19) should be modified by considering the history of the load [44]:

(23) $$\left(2d-2\right)H+\frac{G_{c}}{l_{c}}d-G_{c}l_{c}\Delta d=0$$

where $H=\underset{\left[0,\;t\right]}{\max}\left\{\psi_{+}^{}\right\}$ is a history variable that is the maximum strain energy during the crack propagation. This history variable ensures that the crack face does not close under compression. Equation (23) is the evolution law of the crack phase field for QCs. Cracks grow only if this equation is valid.

### 2.3. Finite Element Implementation

Due to the strong nonlinearity of the governing equations in the phase field model, the FEM is often adopted to solve the problem. In the FEM, the phonon/phason displacements and the phase field variable are approximated by the shape functions:

(24) $${\left\{u_{x},\;u_{y}\right\}}^{T}=\sum_{i=1}^{4}N_{i}^{u}u_{i}$$

(25) $${\left\{w_{x},\;w_{y}\right\}}^{T}=\sum_{i=1}^{4}N_{i}^{w}w_{i}$$

(26) $$d=\sum_{i=1}^{4}N_{i}d_{i}$$

where *N_i_* is the shape function; $N_{i}^{u}=N_{i}^{w}=\mathrm{diag}\left(N_{i},\;N_{i}\right)$; and $u_{i}$, $w_{i}$, and *d* are the nodal phonon and phason displacements and phase field variables, respectively.

Therefore, the phonon and phason strains and the gradient of the phase field variable are, respectively:

(27) $${\left\{\varepsilon_{x},\;\varepsilon_{y},\;\gamma_{xy}\right\}}^{T}=\sum_{i=1}^{4}B_{i}^{u}u_{i}$$

(28) $${\left\{\omega_{x},\;\omega_{y},\;\omega_{xy},\;\omega_{yx}\right\}}^{T}=\sum_{i=1}^{4}B_{i}^{w}w_{i}$$

(29) $$\nabla d=\sum_{i=1}^{4}B_{i}^{d}d_{i}$$

where $B_{i}^{u}={\left[\begin{array}{ccc} \partial N_{i}/\partial x & & \partial N_{i}/\partial y\\ & \partial N_{i}/\partial y & \partial N_{i}/\partial x \end{array}\right]}^{T}$, $B_{i}^{w}={\left[\begin{array}{cccc} \partial N_{i}/\partial x & & \partial N_{i}/\partial y & \\ & \partial N_{i}/\partial y & & \partial N_{i}/\partial x \end{array}\right]}^{T}$, and $B_{i}^{d}={\left[\begin{array}{cc} \partial N_{i}/\partial x & \partial N_{i}/\partial y \end{array}\right]}^{T}$. Substituting Equations (24)–(29) into Equation (15) yields the residuals:

(30) $$r^{a}=\int_{\partial \Omega }^{}N^{T}t\;dS-\int_{\Omega }^{}{\left(1-d\right)}^{2}B^{T}D_{QC}Ba\;dV=0$$

(31) $$r^{d}=-\int_{\Omega }2\left(d-1\right)H^{u}{\left(N^{d}\right)}^{T}\;dV-\int_{\Omega }\frac{G_{c}}{c_{0}}\left[\frac{2d}{l_{c}}{\left(N^{d}\right)}^{T}+2l_{c}{\left(B^{d}\right)}^{T}\nabla d\right]\;dV=0$$

where $a={\left\{u_{x1},\;u_{y1},\;\ldots ,\;u_{x4},\;u_{y4},\;w_{x1},\;w_{y1},\;\ldots ,\;w_{x4},\;w_{y4}\right\}}^{T}$; $N=\left[\begin{array}{cc} N_{0} & 0_{2\times 8}\\ 0_{2\times 8} & N_{0} \end{array}\right]$ is the shape function matrix where $N_{0}=\left[\begin{array}{cccccccc} N_{1} & 0 & N_{2} & 0 & N_{3} & 0 & N_{4} & 0\\ 0 & N_{1} & 0 & N_{2} & 0 & N_{3} & 0 & N_{4} \end{array}\right]$; B=LN is the strain matrix where $L={\left[\begin{array}{ccccccc} \frac{\partial }{\partial x} & 0 & \frac{\partial }{\partial y} & 0 & 0 & 0 & 0\\ 0 & \frac{\partial }{\partial y} & \frac{\partial }{\partial x} & 0 & 0 & 0 & 0\\ 0 & 0 & 0 & \frac{\partial }{\partial x} & 0 & \frac{\partial }{\partial y} & 0\\ 0 & 0 & 0 & 0 & \frac{\partial }{\partial y} & 0 & \frac{\partial }{\partial x} \end{array}\right]}^{T}$; and $t={\left\{t_{x}^{u},\;t_{y}^{u},\;t_{x}^{w},\;t_{y}^{w}\right\}}^{T}$ is the load vector.

Equations (30) and (31) can be solved by this iteration method:

(32) $$\left\{\begin{array}{c} a_{n+1}\\ d_{n+1} \end{array}\right\}=\left\{\begin{array}{c} a_{n}\\ d_{n} \end{array}\right\}+{\left[\begin{array}{cc} K_{n}^{aa} & K_{n}^{ad}\\ K_{n}^{da} & K_{n}^{dd} \end{array}\right]}^{-1}\left\{\begin{array}{c} r_{n}^{a}\\ r_{n}^{d} \end{array}\right\}$$

where $K^{aa}=\int_{\Omega }\omega \left(d\right)B^{T}DB\;dV$, $K^{ad}={\left(K^{da}\right)}^{T}=\int_{\Omega }2\left(d-1\right)B^{T}\sigma N\;dV$, and $K^{dd}=\int_{\Omega }\left(2H+\frac{G_{c}}{l}\right)N^{dT}N^{d}dV+\int_{\Omega }lG_{c}B^{dT}B^{d}dV$.

Due to the high nonlinearity of Equation (32), there is a convergence problem using the classic Newton iteration. A highly robust staggered algorithm is usually adopted to solve Equation (32) [41,44]. In this algorithm, one of the two unknowns (displacement and phase field variable) is assumed to be unchanged while solving the other unknown during one iteration, which yields:

(33) $$a_{n+1}=a_{n}+{\left(K_{n}^{aa}\right)}^{-1}r_{n}^{a}$$

(34) $$d_{n+1}=d_{n}+{\left(K_{n}^{dd}\right)}^{-1}r_{n}^{d}$$

Finally, the nodal phonon/phason displacements and the phase field variables can be calculated by Equations (33) and (34), respectively.

## 3. Numerical Results

In this section, a few numerical examples are presented to illustrate the application of the phase field method to the fracture of 2D decagonal QCs. The material parameters are tabulated in Table 1 [45,46,47].

According to Fan’s work [42], the expression of CERR is:

(35) $$G_{c}=\frac{\lambda \left(K_{1}+K_{2}\right)+2\left(R_{1}^{2}+R_{2}^{2}\right)}{8\left(\lambda +M\right)c}K_{IC}^{2}$$

where $M=\left(C_{11}-C_{12}\right)/2$ and $c=M\left(K_{1}+K_{2}\right)-2\left(R_{1}^{2}+R_{2}^{2}\right)$. The fracture toughness of Al-Ni-Co QCs is $K_{IC}=1\mathrm{MPa}\sqrt{m}$. Therefore, the CERR in this paper is selected as $G_{c}=5.56\times 10^{-4}\;N/\mathrm{mm}$, according to the material constants in Table 1. Although no experimental method has been reported to apply a constant phason displacement on the surface of QCs, some investigations [48,49] reveal that some ways can cause the disorder of the phason field. Therefore, different phason displacement loads are considered in the following calculation to investigate the effect of the phason field on the fracture behavior of QCs.

### 3.1. The Rectangular QCs with Edge Crack

**Figure 2:**
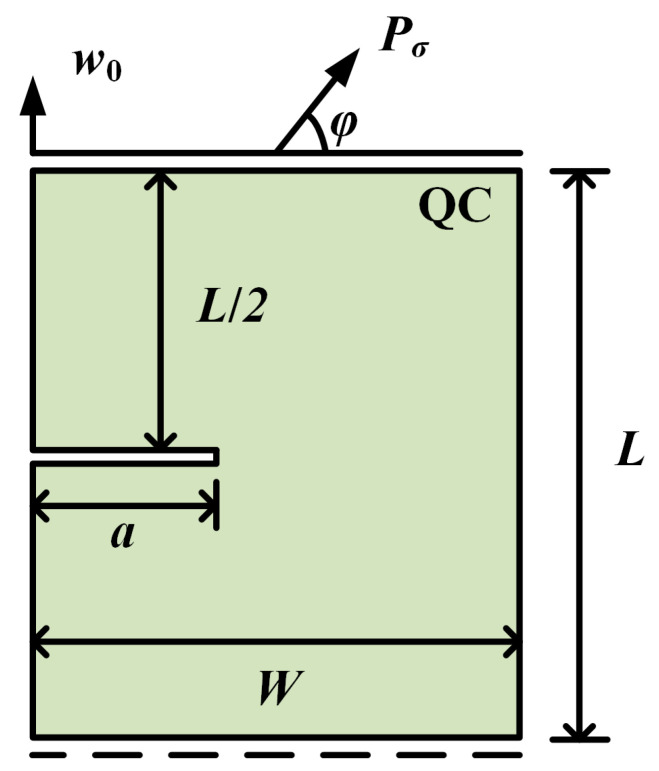
A rectangular QC with an edge crack.

A rectangular QC model is considered, as shown in Figure 2. The geometry of the model is width *W*, height *L*, and crack length *a*. The upper edge is constrained to have the same displacement in phonon and phason fields, and the lower edge is only constrained in the vertical direction. A concentrated phonon force $P_{\sigma }$ is applied to the upper edge. The angle between $P_{\sigma }$ and the horizon is φ.

**Figure 3:**
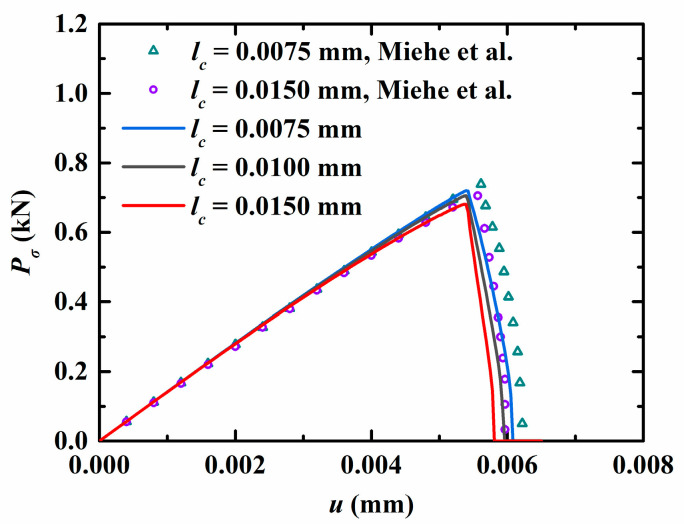
Variation of the force $P_{\sigma }$ versus the displacement *u* [44].

At the current stage, the crack growth of 2D QCs has not been reported in the open literature. To demonstrate the accuracy of the present method, an elastic material is selected by degenerating all the material constants in phason field, i.e., $K_{1}=K_{2}=R_{1}=R_{2}=0$. The geometrical parameters are taken as a=0.5 mm and W=L=1 mm. The elastic material constants come from Ref. [44]. The variation of the vertical concentrated force $P_{\sigma }$ (φ=π/2) versus the displacement *u* is plotted in Figure 3. As observed, the present results are in excellent agreement with the reference data from Ref. [44]. Furthermore, it is noted that the peak force $P_{\sigma }$ shows an increasing trend as the length scale *l_c_* decreases, which indicates the crack grows slowly as the length scale declines. In the previous study, to ensure the high resolution of the crack topology, the minimum size of the element was required to meet the condition of $h<l_{c}/2$ [43]. Therefore, in the following calculations, the length scale is selected as $l_{c}=1\;\mathrm{mm}$, and the element size is selected as $h<l_{c}/5$.

**Figure 4:**
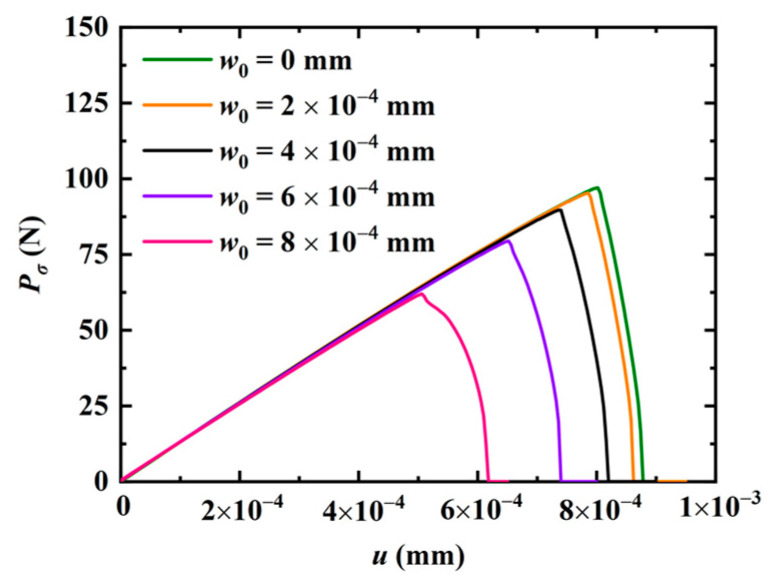
Force-displacement relations for different initial phason loads $w_{0}$.

**Figure 5:**
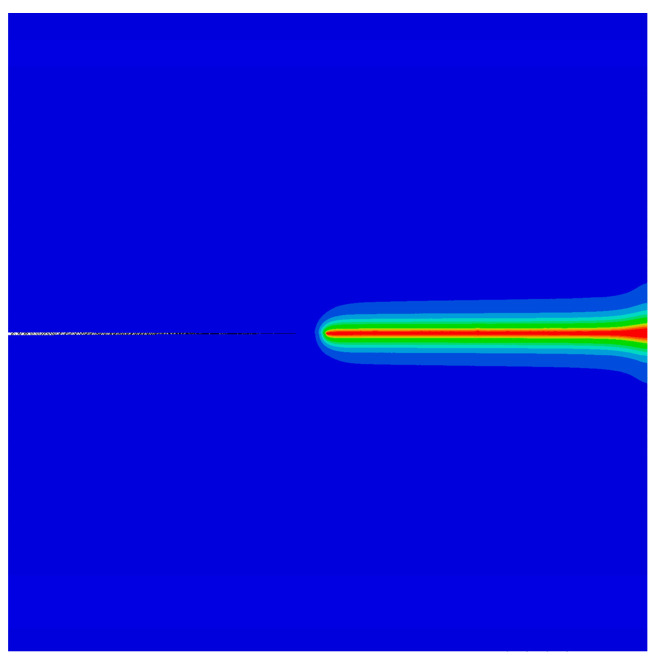
The crack pattern with $w_{0}=6\times 10^{-4}\;\mathrm{mm}$.

Subsequently, the effect of the phason field on the crack growth is studied in Figure 2 by considering an initial phason displacement $w_{0}$ on the upper edge of the model. The parameters are selected as a=50 mm and W=L=100 mm. The variation of the applied force versus the displacement for different $w_{0}$ values is illustrated in Figure 4. The results show that the peak force decreases as the initial phason displacement increases, which indicates that the initial stretch phason load will undermine the strength of the model. In addition, as the initial phason load gets larger, the crack grows slower. The crack pattern with $w_{0}=6\times 10^{-4}\;\mathrm{mm}$ is plotted in Figure 5.

**Figure 6:**
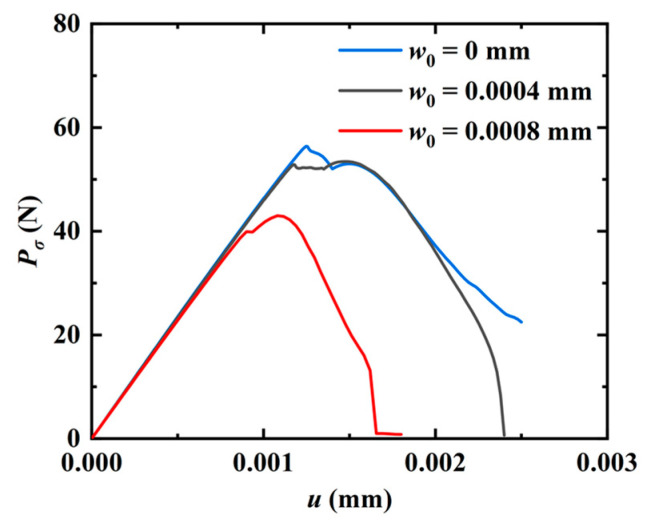
The shear force-displacement relations for different initial tensile phason loads.

**Figure 7:**
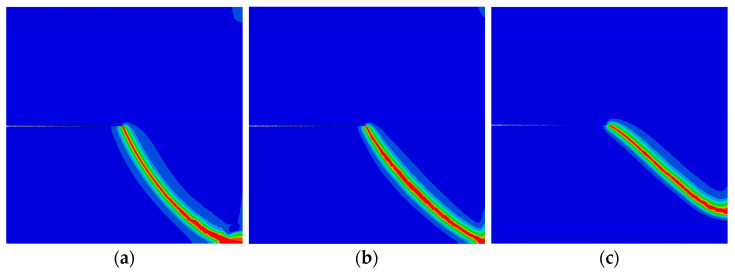
The crack pattern with shear load for different initial phason loads (edge crack): (**a**) $w_{0}=0\;\mathrm{mm}$; (**b**) $w_{0}=4\times 10^{-4}\;\mathrm{mm}$; and (**c**) $w_{0}=8\times 10^{-4}\;\mathrm{mm}$.

Finally, the QC model subjected to a concentrated shear load at its upper edge is considered. The variation of the shear phonon force $P_{\sigma }$ (φ=π) versus the horizontal displacement at the upper edge is illustrated in Figure 6. Two peak forces were observed, and they decrease as the initial vertical phason displacement $w_{0}$ increases. The crack patterns with different initial phason displacements are shown in Figure 7. Clearly, the increasing initial phason displacement will lead to a significant crack deflection. Therefore, it is concluded that the phason load is a key influencing factor for the peak force and crack patterns under the applied shear loads.

### 3.2. The Rectangular QCs with an Internal Crack

**Figure 8:**
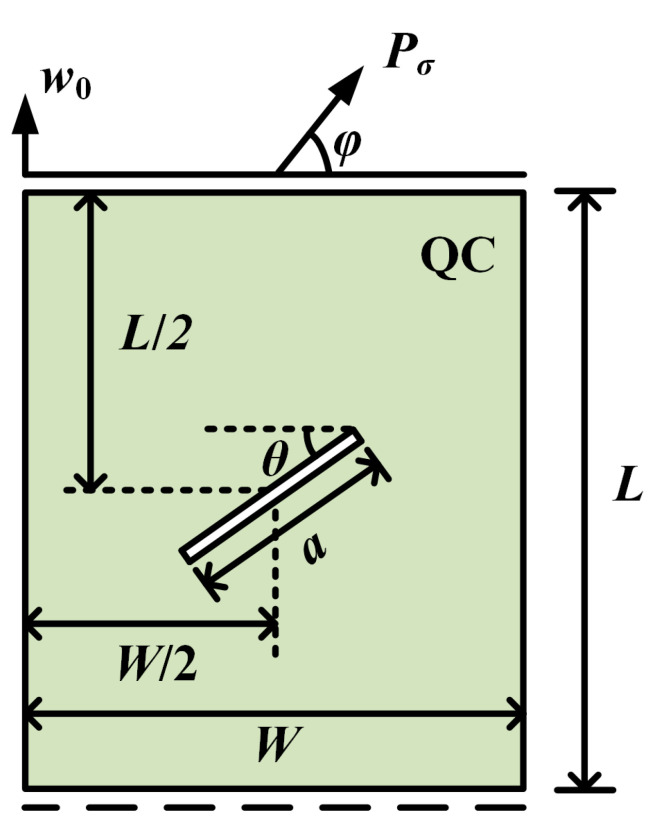
A rectangular QC with an internal crack.

In this example, a rectangular QC with an internal crack is considered in Figure 8. The length of the crack is *a*, and the angle between the crack and the horizon is θ. The middle of the crack is centered in the model. The boundary conditions of the QC are the same as those in Section 3.1. The parameters are selected as a=25 mm and W=L=100 mm. The QC model is subjected to a vertical initial phason displacement $w_{0}$ at the upper edge.

**Figure 9:**
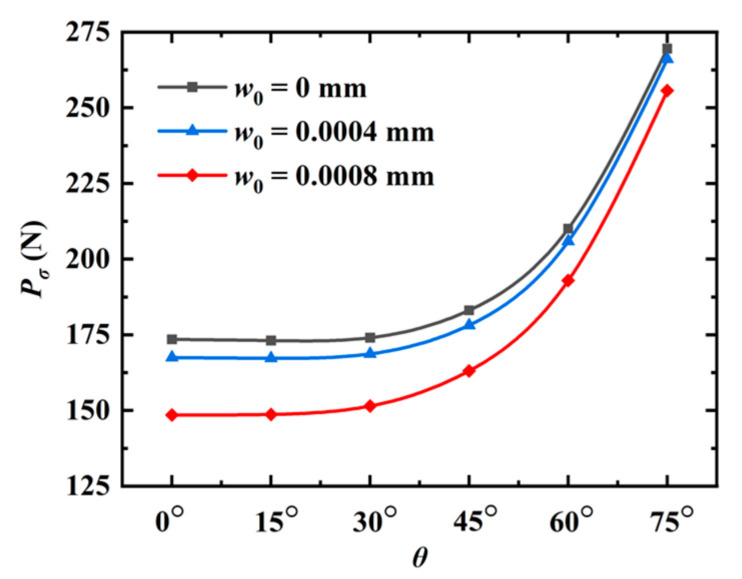
The peak phonon forces for different angles θ (edge crack).

**Figure 10:**
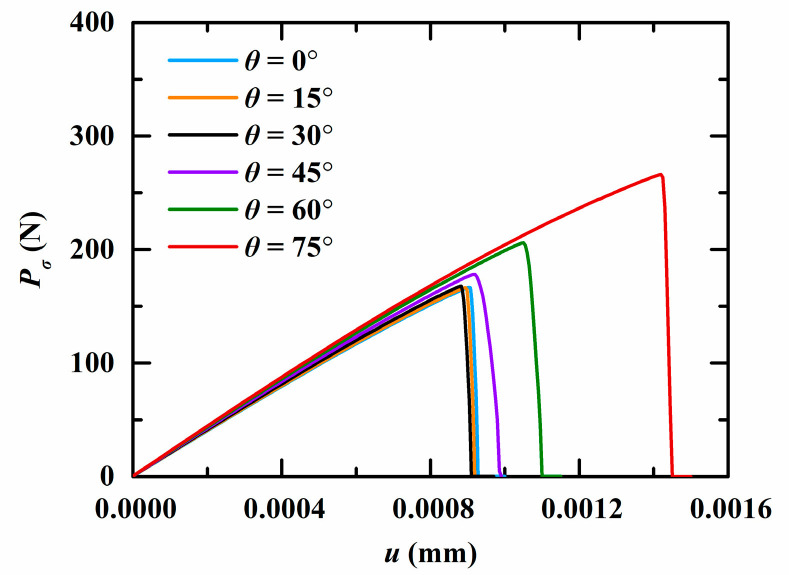
The force-displacement relation for different angles θ.

**Figure 11:**
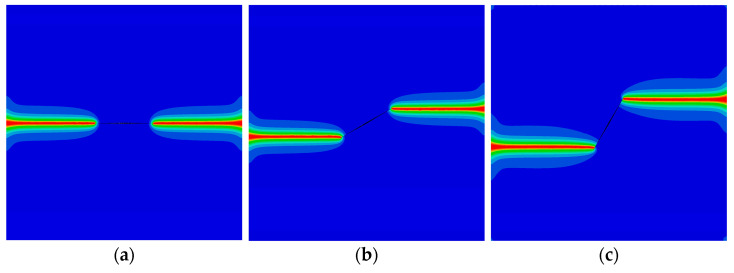
The crack patterns with $w_{0}=6\times 10^{-4}\;\mathrm{mm}$ for different angles θ: (**a**) θ=0; (**b**) θ=π/6; and (**c**) θ=π/3.

Firstly, the QC model is subjected to a tensile load in the phonon field (φ=π/2). The peaks of the phonon forces for different angles θ are shown in Figure 9. It can be observed that the peak force monotonously increases with the increase in θ, while it shows an opposite trend as the initial phason displacement increases. The force-displacement relation for different angles θ is shown in Figure 10. Clearly, the slope of the force-displacement curve increases as the angle θ increases. The crack patterns with different angles are plotted in Figure 11. As observed, the crack grows along a straight line to the edge of the model when subjected to a tensile load.

**Figure 12:**
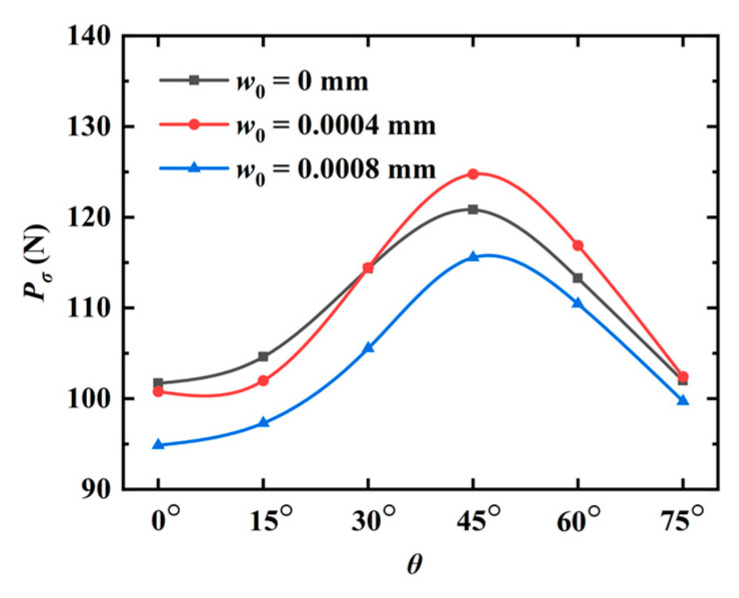
The peak forces for different angles θ (internal crack).

**Figure 13:**
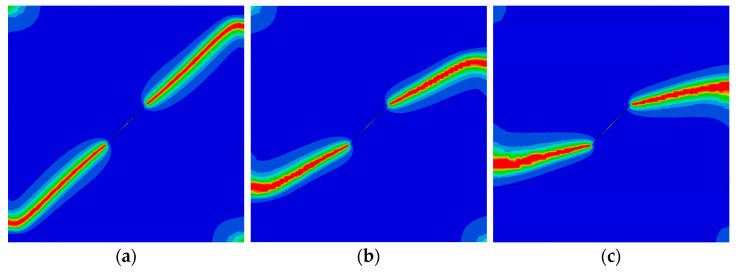
The crack pattern with shear load for different initial phason loads (internal crack): (**a**) $w_{0}=8\times 10^{-4}\;\mathrm{mm}$; (**b**) $w_{0}=1.2\times 10^{-3}\;\mathrm{mm}$; and (**c**) $w_{0}=1.6\times 10^{-3}\;\mathrm{mm}$.

Subsequently, the model is subjected to shear loads in the phonon field at the upper edge. The peak phonon forces for different angles *θ* are illustrated in Figure 12. As observed, the peak force first increases and then decreases as the angle *θ* increases. The crack patterns are plotted in Figure 13. Similar to the observation in Figure 7, the increasing initial phason displacement has a significant influence on the crack propagation path.

### 3.3. The Rectangular QCs with Double Cracks

**Figure 14:**
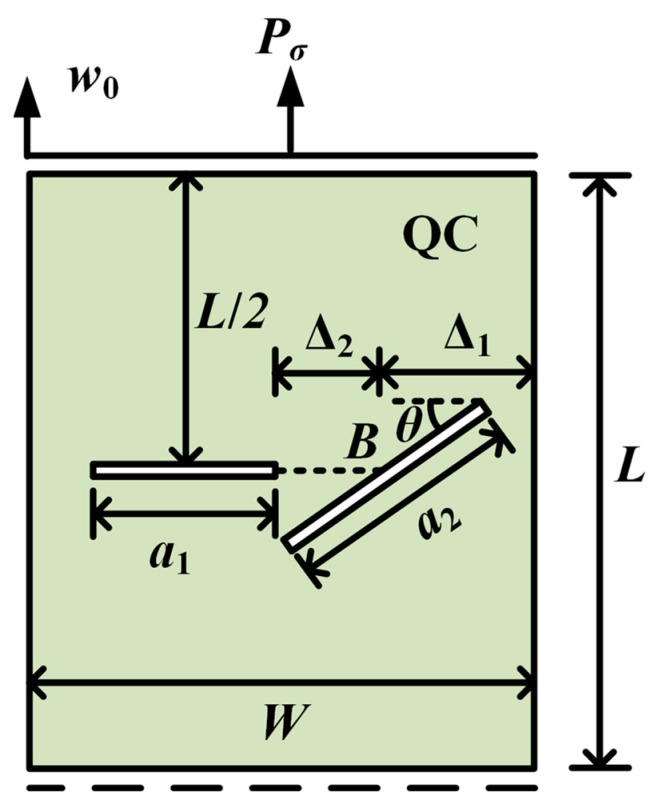
A rectangular QC with double internal cracks.

**Figure 15:**
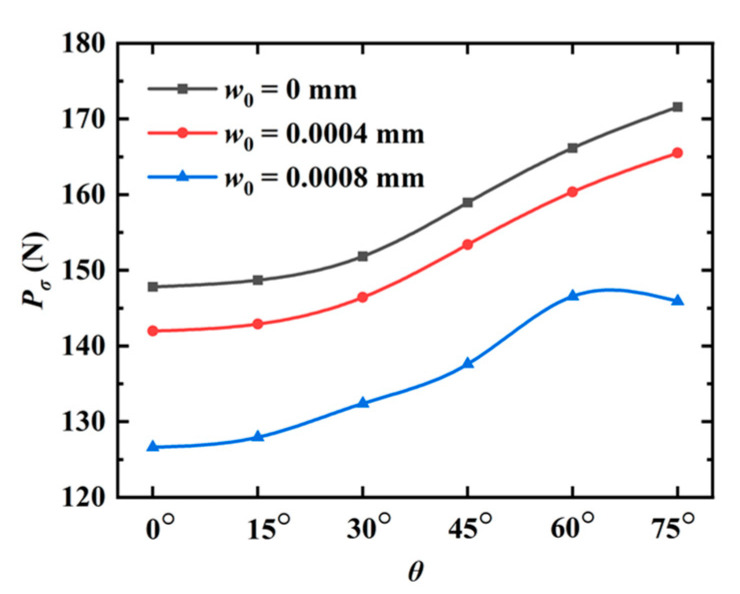
The peak forces for different angles θ (double cracks).

**Figure 16:**
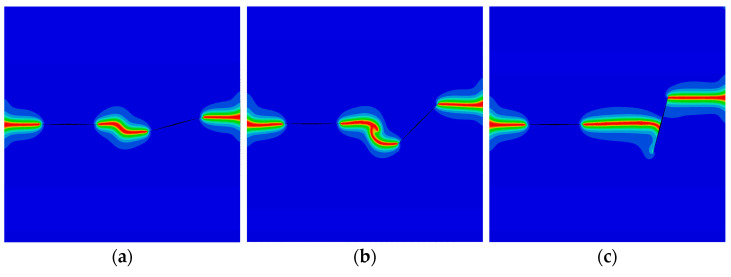
The crack pattern with $\Delta_{2}=32.5\;\mathrm{mm}$ for different angles (double cracks): (**a**) θ=π/12; (**b**) θ=π/4; (**c**) θ=5π/12.

**Figure 17:**
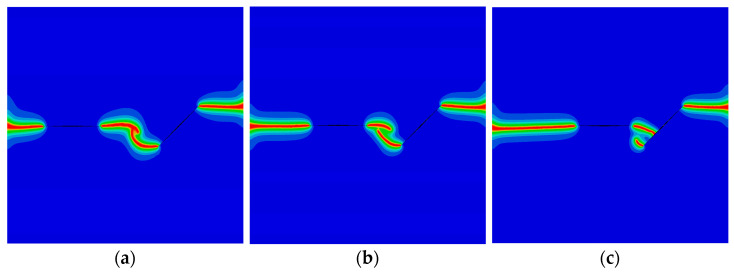
The crack pattern with θ=π/4 for different distances $\Delta_{2}$: (**a**) $\Delta_{2}=32.5\;\mathrm{mm}$; (**b**) $\Delta_{2}=22.5\;\mathrm{mm}$; and (**c**) $\Delta_{2}=12.5\;\mathrm{mm}$.

As the last example, the QC model with double cracks is investigated in Figure 14. The parameters are selected as $a_{1}=a_{2}=25\;\mathrm{mm}$ and W=L=100 mm. The left crack is horizontal. The angle between the right crack and the horizon is θ. The lengths of the left and right cracks are *a*_1_ and *a*_2_, respectively. Point *B* is located at the center of the right crack. The distance between the right edge of the model and point *B* is $\Delta_{1}=27.5\;\mathrm{mm}$. The distance between the left crack and point *B* is $\Delta_{2}$. The model is subjected to a tensile phonon/phason displacements. The peak forces for different angles θ with $\Delta_{2}=32.5\;\mathrm{mm}$ are plotted in Figure 15. It can be found that, as θ increases, the peak force monotonously increases for $w_{0}=0\;\mathrm{mm}$ and $w_{0}=0.0004\;\mathrm{mm}$, while it increases first and then decreases for $w_{0}=0.0008\;\mathrm{mm}$. The crack patterns for different angles θ and distances $\Delta_{2}$ are illustrated in Figure 16 and Figure 17. As observed, the interaction of two cracks has a big contribution to their crack propagation path. The two cracks eventually merge together as the applied load increases.

## 4. Conclusions

In this paper, a phase field model is developed to predict crack propagation in 2D decagonal QCs. The contribution of the phason field to the potential energy is considered, and the evolution law of the crack phase field for QCs is established. Therefore, the crack topology in QCs is described by the phase field variable, which can be solved by the FEM. In this manner, the crack propagation in QCs can be accurately simulated without remeshing, and the evolution of the crack can be vividly illustrated. Numerical examples illustrate that the proposed model can predict accurate results for the crack propagation of QCs, and the phason field has a big contribution to both the force-displacement relation and the crack pattern.

## Figures and Tables

**Table 1:** Material properties of 2D decagonal QCs.

	$C_{11}$	$C_{12}$	$C_{66}$	$K_{1}$	$K_{2}$	$R_{1}$	$R_{2}$
Al-Ni-Co (GPa)	234.30	57.34	88.45	122	24	−1.1	0.1

## Data Availability

The data presented in this study are available on request from the corresponding authors.
